# Puerperal Convulsions Successfully Treated by Subcutaneous Injections of Morphia

**Published:** 1864-10

**Authors:** 

**Affiliations:** Wurtzburg


### PUERPERAL CONVULSIONS SUCCESSFULLY TREATED BY SUBCUTANEOUS INJECTIONS OF MORPHIA.

By PROF. SCANZONI, of Wurtzburg.

Since the attention of the medical profession was first directed by Dr. Wood, of Edinburgh, and more lately by Hunter and Behier, to the advantageous effects of subcutaneous injection, especially of narcotics, Professor Scanzoni has employed this method with success in numerous cases of neuralgia, hyperæsthesia, etc.; but he attaches especial importance to the following case of puerperal convulsions, because it seems to prove, in accordance with the views laid down by Hunter, that the subcutaneous application of narcotic agents furnishes a means of acting on abnormal irritations of the brain with greater rapidity and certainty than the administration of the same remedies by the mouth. It will, doubtless, be admitted that opium, in its different preparations, deserve the first place in the treatment of puerperal eclampsia. In his own experience, the observation of a large number of cases has convinced Professor Scanzoni that a kind of intoxication produced by opium leads with more certainty to a favorable termination than any other means recommended in this terrible disease. But, unfortunately, it is not always possible to administer a sufficient quantity of opium or morphia; sometimes the comatose condition of the patient, at other times the rapid succession of paroxysms, prevents administration by the mouth; and opiate enemata are occasionally rejected as soon as they are received. The subcutaneous injection, however, supplies the means by which these difficulties may be overcome, and a sufficient quantity of opium introduced into the system to render its effects certain. Numerous experiments have convinced the author that, although the effect of this method is not always persistent (the neuralgiæ, for example, are not always cured by it), yet there are constantly produced—within a very short time, often a few minutes, after the injection—certain phenomena, which can leave no doubt as to the action of the opium upon the brain. Such symptoms are drowsiness, giddiness, headache, sickness, feeling of constriction in the throat, even vomiting, and depression; or, if the dose is large, somnolence. These facts, taken along with the known effects of the subcutaneous application in delirium tremens, mania, chorea, tetanus, etc., induced him to try the same treatment in puerperal convulsions, and with the most satisfactory results. After three injections of the meconate of morphia there occurred only two attacks in nine hours, while previously there had been three attacks in an hour and three quarters. This diminution of the convulsions after the injections is so much the more remarkable, since experience has shown that, as a general rule, the paroxysms become not only more violent, but follow at shorter intervals as the labor advances. And although the author does not imagine that he has ddiscovered in the subcutaneous injection an infallible panacea for this dreadful malady, he is of opinion that the following case should induce physicians to give this means a trial:

*Case*—D., aged 21, primipara, strong and robust, was brought into the lying-in ward at a quarter to eight o'clock on the morning of June 8th, 1859. Labor had commenced in the night, and she had been seized with nervous paroxysms and loss of consciousness; no account was given of the nature of the attacks; the patient remembered nothing of what had occurred during the night. The whole body, and especially the lower extremities, were odematous; on the right side of the tongue showed marks of being bitten by the teeth; the uterus corresponded to the pit of the stomach, and seemed sufficiently consistent; sounds of the fœtal heart distinct. On examination, the os uteri was dilated to the size of a sixpence, the bag of waters was partly formed, and the head presented; the urine was very albuminous, and exhibited under the microscope numerous fibrinous cylinders. At eight o'clock she was seized with a second convulsive attack, which was of a very marked character, and lasted some minutes. On recovering consciousness, she could answer questions, although slowly. A third attack succeeded at a quarter to nine, a fourth at a quarter to ten, a fifth at a quarter to twelve, and a sixth at five o'clock—the last the most violent. After the fourth paroxysm consciousness did not return, and the breathing became stertorous. At ten o'clock she was bled to about eight ounces, an enema with twenty-five drops of laudanum was given, the body was put into a warm bath, while cold irrigation was applied to the head. As opium could not be administered internally, a solution of the meconate of morphia was now, at three different times, injected under the skin, the quantity amounting in all to about ten grains (seventy-five centigrammes) of opium. The labor advanced very slowly. At three o'clock next morning the membranes burst; the os dilated to the size of a half crown; the head still high up above the brim; sounds of the heart very distinct. After this period the dilatation went on more quickly; at seven o'clock the os was larger than a crown piece, very extensive and dilatable, the head high up and immovable; complete loss of consciousness, profound coma. In these circumstances, which left little hope of saving the patient, and in spite of the high position of the head and the incomplete dilatation of the os uteri, it was decided to employ the forceps. Their application was by no means easy, but the extraction presented no difficulty. After a few tractions, a fœtus was born, which breathed feebly at first, but soon began to moan vigorously; the placenta followed. During the operation there was no paroxysm. Some wine and ten drops of tincture of amber and musk were now given to the patient, which revived her a little, but did not restore consciousness. At eleven o'clock, a seventh attack came on, but was slight and short; after which she became excited and tried to escape, but towards morning she grew calm. At nine in the morning she could answer questions put with a loud voice. During the whole day she remained like a drunk person; pulse 128. The musk was stopped; nothing but lemonade given. Towards evening the abdomen was somewhat painful. During the night there were several slight attacks of mania; she constantly attempted to escape. In the morning she answered rationally; pulse 108. The œdema had diminished, the abdomen was still tender; there was difficulty of breathing; and numerous râles, fine and coarse, in the lungs. Warm bath, lemonade, expectorants, were prescribed. In the evening the patient was completely herself again; pulse 132. June 11th and 12th—She slept well during the night, the expectoration becoming easy, and the pain of the abdomen relieved by fomentations and poultices; pulse 120; the urine contained little albumen, and no fibrinous cylinders. June 13th—Good condition; œdema gone; abdomen soft; some incontinence of urine during the night, was relieved by leaving in a catheter. All medicines were now suspended; the patient was put on good diet, and ordered to take every morning a glass of chalybeate mineral water. On the 17th there was no albumen found in the urine, and on the 21st the patient left the hospital with her child, being advised to continue the use of steel for a considerable time.

### THE LA POMMERAIS CASE.

A homœopathic practitioner of Paris, named La Pommerais, has been lately condemned to death for the murder of his mistress, who he had previously induced to insure her life in different offices to the amount of one hundred and ten thousand dollars. The payment of life insurance, in this case, has given rise to very interesting medico-legal questions.

First, it may be asked whether the sum for which the life of the victim of the charlatan Pommerais was insured, will have to be paid over to her children, and, secondly, whether the transfers executed by her in favor of La Pommerais are valid, and his representatives will have any claim on the companies. The insurances, it is contended, will be available for the children of the murdered woman It is established, and the companies have not, it is believed, disputed the fact, that they did not sign the contracts drawn up in her name and for her behalf without having taken all the preliminary steps usual in such cases, and obtained all the information respecting the insurer when they covenanted to pay a specified sum. The conditions of the contract between Madame de Pauw and the companies were fulfilled so far. She had been visited by the companies' medical officers, who reported that her health was excellent, and that the insurance might be safely effected; and the first premium was punctually paid. The policies of insurance specify only two cases were insurance companies may refuse payment—suicide or death in a duel; but when the party insured is murdered, his or her heirs must not suffer by the crime of a third party, nor are the companies released from their engagements. It was the woman's life that was insured, and not La Pommerais's; and in no case would the latter make anything by the transfer of her title to him, as it was shown to be obtained by fraud.

